# Redox imbalance in patients with heart failure and ICD/CRT-D intervention. Can it be an underappreciated and overlooked arrhythmogenic factor? A first preliminary clinical study

**DOI:** 10.3389/fphys.2023.1289587

**Published:** 2023-11-08

**Authors:** Jakub Szyller, Radosław Antoniak, Katarzyna Wadowska, Iwona Bil-Lula, Bruno Hrymniak, Waldemar Banasiak, Dariusz Jagielski

**Affiliations:** ^1^ Division of Clinical Chemistry and Laboratory Hematology, Department of Medical Laboratory Diagnostics, Faculty of Pharmacy, Wroclaw Medical University, Wroclaw, Poland; ^2^ Department of Cardiology, Centre for Heart Diseases, 4th Military Hospital, Wroclaw, Poland; ^3^ Faculty of Medicine, Wroclaw University of Science and Technology, Wroclaw, Poland

**Keywords:** oxidative stress, redox imbalance, ventricular arrhythmia, implantable cardioverter-defibrillator, cardiac resynchronization therapy, heart failure

## Abstract

**Introduction:** Redox imbalance and oxidative stress are involved in the pathogenesis of arrhythmias. They also play a significant role in pathogenesis of heart failure (HF). In patients with HFand implanted cardioverter-defibrillator (ICD) or cardiac resynchronization therapy defibrillator (CRT-D), the direct current shocks may be responsible for additional redox disturbances and additionally increase arrhythmia risk. However, the precise role of oxidative stress in potentially fatal arrhythmias and shock induction remains unclear.

**Methods:** 36 patients with diagnosed HF and implanted ICD/CRT-D were included in this study. Patients were qualified to the study group in case of registered ventricular arrhythmia and adequate ICD/CRT-D intervention. The control group consisted of patients without arrhythmia with elective replacement indicator (ERI) status. Activity of superoxide dismutase (SOD), catalase (CAT), glutathione peroxidase (GPx), glutathione (GSH) in erythrocyte (RBC), SOD, GPx activity and reactive oxygen/nitrogen species (ROS/RNS) concentration in plasma were determined. The values were correlated with glucose, TSH, uric acid, Mg and ion concentrations.

**Results:** In the perishock period, we found a significant decrease in RBC and extracellular (EC) SOD and RBC CAT activity (*p* = 0.0110, *p* = 0.0055 and *p* = 0.0002, respectively). EC GPx activity was also lower (*p* = 0.0313). In all patients, a decrease in the concentration of all forms of glutathione was observed compared to the ERI group. Important association between ROS/RNS and GSH, Mg, TSH and uric acid was shown. A relationship between the activity of GSH and antioxidant enzymes was found. Furthermore, an association between oxidative stress and ionic imbalance has also been demonstrated. The patients had an unchanged de Haan antioxidant ratio and glutathione redox potential.

**Conclusion:** Here we show significant redox disturbances in patients with HF and ICD/CRT-D interventions. Oxidative stress may be an additional risk factor for the development of arrhythmia in patients with HF. The detailed role of oxidative stress in ventricular arrhythmias requires further research already undertaken by our team.

## 1 Introduction

Oxidative stress is defined as an imbalance between the production and accumulation of reactive oxygen species (ROS) in cells and tissues. The inability of biological systems to detoxify these reactive products leads to a disruption of redox signaling and control and/or molecular damage (Jones and Sies, 2015). This phenomenon involves the pathogenesis of many cardiovascular diseases, including cardiac arrhythmias (Tanaka et al., 2011; Sovari et al., 2013; Kazemirad and Kazerani, 2020; Liu et al., 2021; Xie et al., 2021; Szyller et al., 2022). The main sources of cardiac ROS are mitochondria, NADPH oxidase and uncoupling of endothelial nitric oxide synthase (Tsutsui et al., 2011; Liang et al., 2020). There are several ways for ROS to induce cardiac arrhythmias. ROS, apart from other known factors, such as infection, hypoxia, etc., can increase focal myocardial electrical activity by promotion of an early afterdepolarizations mechanism (Morita et al., 2009; Karagueuzian et al., 2013), modification of substrate for reentry (Morita et al., 2011; Sovari et al., 2011; Karagueuzian et al., 2013) or by affecting several ionic currents in cardiomyocytes (Annunziato et al., 2002). The arrhythmogenic potential may result from Ca^2+^ release and enhance inadequate electrical activity (Lin et al., 2010), the Ca^2+^-ATPase pump inhibition and the Ca^2+^ release channels activation (Favor et al., 1995). Redox imbalance, such as decreased glutathione peroxidase (GPx) activity (Bezna et al., 2022), decreased ratio of reduced to oxidized glutathione (GSH/GSSG), reduced to oxidized cysteine (Cys/CysS), NADPH oxidase activation (Neuman et al., 2007) or superoxide (O_2_^•-^) formation is independently associated with cardiac arrhythmias (Kim et al., 2005; 2008).

Enzymatic and non-enzymatic antioxidants act to suppress or prevent the formation of ROS and are the principal components of the defense system. Three key first-line enzymes are superoxide dismutase (SOD), catalase (CAT) and GPx. The second line of defense antioxidants is often referred as scavenging antioxidants. They can neutralize or scavenge free radicals. Members of the non-enzymatic antioxidants are, e.g.,: ascorbic acid, alpha-tocopherol, uric acid (UA) or glutathione (GSH). Oxidative stress biomarkers such as the activity of antioxidant enzymes and total antioxidant capacity or ROS concentration can correlate with the occurrence of arrhythmias (Türker et al., 2016; Yang et al., 2018; Adameova et al., 2020; Michałek et al., 2020).

So far, numerous studies link oxidative stress to the most common supraventricular arrhythmia - atrial fibrillation (AF) (Shite et al., 2001; Neuman et al., 2007; Lin et al., 2010; Xie et al., 2015; Avula et al., 2021). Little is known about its role in the development of ventricular tachycardia (VT), ventricular fibrillation (VF) or other ventricular arrhythmias (VAs). VT and VF are the most common life-threatening arrhythmias leading to sudden cardiac death (Kauppila et al., 2018). It is a serious medical problem for many patients, among others, with hereditary arrhythmia syndromes (especially in young patients), coronary artery disease, postoperative arrhythmias or with heart failure (HF). In the latter group of patients, oxidative stress is an important pathogenic factor in developing and progressing of HF and arrhythmias (Dai et al., 2011; Romuk et al., 2019; van der Pol et al., 2019). Implantable cardioverter-defibrillators (ICDs) or cardiac resynchronization therapy defibrillators (CRT-D) are key components of the management of patients with HF with reduced left ventricular ejection fraction (LVEF) (indicated for primary or secondary prevention). Electric direct current (DC) shock can also be a source of free radicals that favor the development of cardiac arrhythmias. The exact role of oxidative stress in the development of arrhythmias in these patients has not been recognized so far.

Therefore, this study aims to evaluate the SOD, CAT and GPx activity, ROS/RNS and GSH concentrations in patients with VAs and ICD/CRT-D interventions. To our knowledge, this is the first study in patients with HF which evaluates the redox status in the period around the ICD/CRT-D adequate interventions.

## 2 Materials and method

### 2.1 Participants

The study population consisted of 36 patients with diagnosed HF and implanted ICD or CRT-D devices. The inclusion and exclusion criteria were clearly defined at the study design level (Table 1). All patients understood their participation in the research study and recognized its purpose. Written informed consent was obtained from all participants. The study was approved by the Bioethical Committee (KB 212/2023) and is in compliance with the Helsinki Declaration (1964) and its later amendments. The general characteristics of the groups are presented in Table 2.

**TABLE 1:** Detailed inclusion and exclusion criteria in the study.

Inclusion criteria		Exclusion criteria
Intervention group	Control group (ERI)	
1. Written consent of the patient to participate in the study		1. No possibility of obtaining written consent for the study
2. Diagnosed HF		2. Acute HF decompensation
		3. The presence of comorbidities such as thyroid disease, cancer, HIV infection, viral hepatitis, valvular disease
3. Registered ventricular arrhythmia and ICD/CRT-D shock (adequate intervention)	6. Alcohol abuse (acceptable occasional consumption), use of psychoactive substances or stimulants	
	3. Qualification for ICD/CRT-D device replacement (positive Elective Replacement Indicator (ERI) status)	4. Active inflammation or infection (e.g., myocarditis, pneumonia, pancreatitis, recent tooth extraction and dental treatment)
4. Absence of acute inflammation confirmed by CRP concentration measurement (serum CRP <15 mg/L)	4. No ICD intervention min In the previous 3 months7	
		5. Smoking (including e-cigarettes)

5. No clinical features of HF decompensation		

**TABLE 2:** General characteristics of the study groups.

	Intervention group	Control group	Intervention vs. control, *p-value*
Number of patients	16	20	
Age (years)	60.8 ± 3.3 (median: 68.0)	74.4 ± 2.0 (median: 74.0)	**0.0072**
Sex (male/female)	14/2	16/4	0.8808
Etiology of heart failure			
ischemic	69% (n = 11)	65% (n = 13)	0.9056
non-ischemic	31% (n = 5)	35% (n = 7)	
LVEF (%)	38.3 ± 12.7	37.0 ± 11.4	0.8019
NYHA			
I	56% (n = 9)	15% (n = 3)	0.1237
II	31% (n = 5)	65% (n = 13)	0.0935
III	13% (n = 2)	20% (n = 4)	0.8808
IV	---	---	
Arrhythmia			
VT	38% (n = 6)	---	---
VF	31% (n = 5)		
ES	31% (n = 5)		
**Laboratory tests**			
Na (mmol/L)	139.5 ± 2.3	141.9 ± 2.7	**0.0060**
K (mmol/L)	4.1 ± 0.4	4.2 ± 0.3	0.1403
Mg (mmol/L)	0.87 ± 0.10	0.87 ± 0.08	0.7278
TSH (µIU/mL)	2.114 ± 1.704	2.176 ± 0.946	0.8944
Creatinine (mg/dL)	1.14 ± 0.40	1.14 ± 0.33	0.9949
CRP (mg/L)	3.70 ± 4.11	2.53 ± 2.45	0.7480
Glucose (mg/dL)	129.2 ± 42.2	114.2 ± 25.3	0.1933
**Comorbidities**			
Hypertension	69% (n = 11)	85% (n = 17)	0.2439
Diabetes mellitus	19% (n = 3)	60% (n = 12)	**0.0312**
Hypercholesterolemia	69% (n = 11)	70% (n = 14)	0.7771
**Classes of drugs**			
ACEi	63% (n = 10)	65% (n = 13)	0.8462
Diuretics	63% (n = 10)	85% (n = 17)	0.2453
Beta-blockers	81% (n = 13)	95% (n = 19)	0.4408
Statins	69% (n = 11)	90% (n = 18)	0.2392
ARB	19% (n = 3)	25% (n = 5)	0.9643
Gliflosins	25% (n = 4)	20% (n = 4)	0.9643
Acetylsalicylic acid	19% (n = 3)	25% (n = 5)	0.9643
VKA	69% (n = 11)	45% (n = 9)	0.4397
Clopidogrel	6% (n = 1)	0% (n = 0)	0.9304

LVEF, left ventricle ejection fraction; NYHA, New York Heart Association; VT, ventricular tachycardia; VF, ventricular fibrillation; ES, electrical storm; TSH, thyroid stimulating hormone; CRP, C–reactive protein; ACEi, angiotensin–converting enzyme inhibitors; ARB, angiotensin 2 receptor blockers; VKA, vitamin K antagonists.

### 2.2 Blood collection

The blood samples were drawn up to max. 6 h after discharge of ICD/CRT-D from a median cubital vein or cephalic vein into a tube containing dipotassium salt of ethylenediaminetetraacetic acid (EDTA-K_2_) (Cat. no. 454023, Vacuette^®^ K_2_EDTA 4 mL, Greiner Bio-one) to obtain erythrocytes and plasma and in a tube with a clot activator (Cat. no. 454092, Vacuette^®^ Tube 4 mL CAT Serum Clot Activator, Greiner Bio-one) to obtain serum. The time of sample collection was due to the delay related to patient’s admission to the hospital or brought by the Emergency Medical Service (EMS). Both samples were transported to the clinical laboratory in a cooler with an ice block within max. 30 min of being drawn, then centrifuged at 2,000 x g for 10 min at 4 °C and finally placed on ice. Plasma or serum has been transferred to the polypropylene microtubes (Eppendorf, Hamburg, Germany) and stored until use at −80 °C.

#### 2.2.1 Preparation of erythrocyte lysate

The blood collected in EDTA-K2 tubes was centrifuged at 2,000 x g for 10 min at 4 °C. 500 μL of pelleted red blood cells were collected from the bottom of the tube into a separate tube and 2 mL of cold phosphate-buffered saline (PBS) was added. Then, the sample was centrifuged at 300 *g* for 5 min. The supernatant was discarded, and 2 mL of PBS was added again. Washing was performed a total of 4 times. After removing the supernatant, 2 mL of ice-cold deionized water was added to the erythrocytes and left for 5 min at 4 °C. After centrifugation at 10,000 x g for 10 min at 4 °C, the lysate was transferred to 1.5 mL microtubes (Eppendorf, Hamburg, Germany) and stored at −80 °C until analysis, but not longer than 1 month.

### 2.3 Determination of hemoglobin concentration

Hemoglobin concentration in erythrocyte lysate was measured spectrophotometrically by the cyanomethemoglobin method (Drabkin-Austin method) using Drabkin’s reagent. In this method, the hemoglobin in the sample is converted to cyanmethemoglobin. The working solution was prepared by dissolving 0.61 mM potassiumhexacyanoferrat III) (cat. no. 244023, Sigma-Aldrich, Darmstadt, Germany), 0.77 mM potassium cyanide (cat. no. 104967, Merck Milipore, United State), 11.9 mM sodium bicarbonate (cat. no. 118105307, Chem-Pur, Poland) in 1,000 mL of deionized water. The solution was kept in a dark bottle all the time. In a polystyrene cuvette containing 2.5 mL of Drabkin’s reagent, 10 μL of erythrocyte lysate was added and the absorbance was read after 5 min against the reagent blank at a wavelength of 540 nm using an UV-Vis spectrophotometer (Marcel Media, Marcel SA, Poland). All samples were measured in duplicate. The results are shown in g/dL.

### 2.4 Measurement of superoxide dismutase activity

SOD (EC 1.15.1.1) activity in the erythrocytes lysates (Cu-ZnSOD, SOD1) and plasma samples (extracellular, EC SOD) was measured with the help of Superoxide Dismutase Assay Kit (Item no. 706002, Cayman Chemicals, Ann Arbor, MI, United States ), that uses tetrazolium salt for detection of superoxide radical. It is produced by xanthine oxidase and hypoxanthine. Collected lysates were diluted 100-fold, plasma 5-fold and analyzed following the assay protocol. Absorbance was measured at 450 nm (Spark Multimode Microplate Reader, Tecan Trading AG, Switzerland). All measurements were performed in duplicate. Units of SOD activity were calculated from a standard curve using purified bovine erythrocyte SOD enzyme. The activity of SOD in plasma was expressed as U/mL. One unit of SOD activity is defined as the amount of enzyme needed to dismutase 50% of available superoxide radicals. The activities of SOD in erythrocytes lysates were converted to grams of hemoglobin and expressed as U/g Hb.

### 2.5 Measurement of catalase activity

Catalase (EC 1.11.1.6) activity in erythrocytes lysates was determined by the UV spectrophotometric method of Aebi (1984) and was carried out in the Laboratory of Elemental Analysis and Structural Research of Wroclaw Medical University. Each of the samples of erythrocyte lysate were diluted 501-fold (10 µL sample was mixed with 5 mL of phosphate buffer and was kept on ice). Sodium, potassium phosphate buffer (50 mM, pH 7.4) was prepared by dissolving 6.81 g of KH_2_PO_4_ (Cat. no. 117420202, Chem-Pur, Poland) and 8.90 g of Na_2_HPO_4_ (Cat. no. 117992801, Chem-Pur, Poland) in 1,000 mL distilled water. The solutions were mixed in the proportion 1:1.5 (v/v). In a quartz cuvette containing 1,000 μL of diluted erythrocyte lysate, 500 μL of 20 mM H_2_O_2_ in phosphate buffer was added and the decrease in absorbance was read at 240 nm for 30 s in a spectrophotometer (UV-Vis Double Beam HALO DB-20 Spectrofotometer, Dynamica GmbH, Switzerland). The 20 mM H_2_O_2_ solution was freshly prepared from 3% H_2_O_2_ (Galfarm, Poland) and standardized using a molar extinction coefficient (*ε* = 43.6 M^–1^ cm^–1^ at 240 nm). All samples were assessed in triplicate. One unit of catalase will decompose 1.0 μmol of hydrogen peroxide to oxygen and water per minute at pH 7.0 at 25 °C at a substrate concentration of 20 mM hydrogen peroxide. The activity of ACT in erythrocyte lysate was converted to grams of hemoglobin and expressed as kU/g Hb. CAT was determined only in erythrocytes, mainly as an intracellular enzyme.

### 2.6 Analysis of glutathione peroxidase activity

GPx (EC 1.11.1.9) activity in the erythrocytes lysates (mainly GPx1) and plasma samples (GPx3) was measured with Glutathione Peroxidase Assay Kit (Item no. 703102, Cayman Chemicals, Ann Arbor, MI, United States ). The test measures the decrease in absorbance at a wavelength of 340 nm during oxidation of NADPH to NADP+, which is formed upon reduction of oxidized glutathione (GSSG) to its reduced state (GSH) by glutathione reductase. The rate of decrease in the absorbance is directly proportional to the GPx activity in the sample. As the substrate cumene hydroperoxide was used to avoid the impact of the heme peroxidase activity of hemoglobin in erythrocyte lysate, which falsely elevated GPx activity. The activities of GPx in erythrocyte lysate were converted to grams of hemoglobin and expressed as U/g Hb (one unit is defined as the amount of enzyme that will cause the oxidation of 0.1 nmol of NADPH to NADP^+^ per minute at 25 °C).

### 2.7 Determination of total glutathione, GSH, GSSG and redox state

Glutathione Assay Kit (Item no. 703002, Cayman Chemical Company, Ann Arbor, MI, United States) was used to measure the total glutathione (GSH + GSSG) and oxidized glutathione (GSSG) levels in erythrocytes. The reaction between sulfhydryl groups of GSH and Ellman’s reagent (DTNB, 5,5′-dithio-bis-2-nitrobenzoic acid) results in a yellow-colored product TNB (5-thio-2-nitrobenzoic acid). Quantification of GSSG was preceded by derivatization of GSH with 2-vinylpyridine (Item no. 132292, Sigma-Aldrich, St. Louis, MO, United States) for the chemical masking of GSH. 2-vinylpyridine alkylates glutathione at slightly acidic pH values where spontaneous formation of glutathione disulfide is minimal. Samples for GSH determination were diluted 1000-fold, and for GSSG determination 10-fold. Due to the fact that 2-vinylpyridine inhibits color development in the assay, two separate standard curves were made for GHS and GSSG. The absorbance of TNB was measured at 410 nm by a microplate reader (Spark Multimode Microplate Reader, Tecan Trading AG, Switzerland). All measurements were performed in duplicate. The total glutathione, GSH and GSSG concentration was expressed in μM/g Hb. The redox state (E_h_) of thiol/disulfide pools was calculated using the Nernst equation (E_h_ = E_o_+(RT/2F)ln [(GSSG)/(GSH)^2^], where E_0_ is the electrochemical force under standard condition (a constant characterizing the redox reaction under consideration), R is the gas constant (8,314 JK^−1^mol^−1^), T the absolute temperature, n the number of electrons involved (n = 2), F the Faraday constant (96,485 Cmol^−1^). E_h_ GSH was expressed as redox potential in millivolts (mV).

### 2.8 Assessment of oxidative stress (ROS/RNS concentration)

OxiSelect™ *In Vitro* ROS/RNS (Green Fluorescence) Assay Kit (Cell Biolabs, San Diego, United States ) was used to determine the oxidative stress in plasma. The assay measures total ROS and reactive nitrogen species (RNS), including hydrogen peroxide (H_2_O_2_), nitric oxide (NO), peroxyl radical (ROO^•^), and peroxynitrite anion (ONOO^−^), using a fluorogenic probe - dichlorodihydrofluorescin DiOxyQ (DCFH-DiOxyQ). The probe is primed with a quench removal reagent to the highly reactive DCFH form. In this reactive state and in the presence of ROS and RNS, the DCFH is rapidly oxidized to the highly fluorescent 2′,7′-dichlorodihydrofluorescein (DCF). The fluorescence of blank samples was subtracted from sample measurements to eliminate background fluorescence. Fluorescence intensity (reading: 480 nm and emission: 530 nm, Spark Multimode Microplate Reader, Tecan Trading AG, Switzerland) is proportional to the total ROS/RNS level within the sample.

### 2.9 Statistical analysis

The experimental data were analysed using GraphPad Prism 8.0.1 for Windows (GraphPad Software, San Diego, California, United States). The Shapiro-Wilk normality test was used to assess the normality of a dataset. For comparisons between two independent groups of measurements of normally distributed data the Student’s t-test was used. The data without normal distribution were compared with the U Mann-Whitney test. In the case of unequal variances, the *t*-test with Welch correction was used. The correlations between quantitative variables were assessed with the Pearson’s (when the data was normally distributed) or Spearman’s correlation coefficient (otherwise). The strength of association was judged with the following scores: I) |r| ≥ 0.9—very strong, II) 0.7 ≤ |r| < 0.9—strong, III) 0.5 ≤ |r| < 0.7—moderate, IV) 0.3 ≤ |r| < 0.5—weak. V) |r| < 0.3—very weak. Yates’s Chi-squared test was used to compare categorical variables between the groups. Results were expressed as mean ± standard error of the mean (SEM) or as median with interquartile range (IQR) according to distribution. *p*-value of <0.05 was regarded as statistically significant.

## 3 Results

### 3.1 Activity of erythrocyte and plasma antioxidant enzymes

**FIGURE 1:**
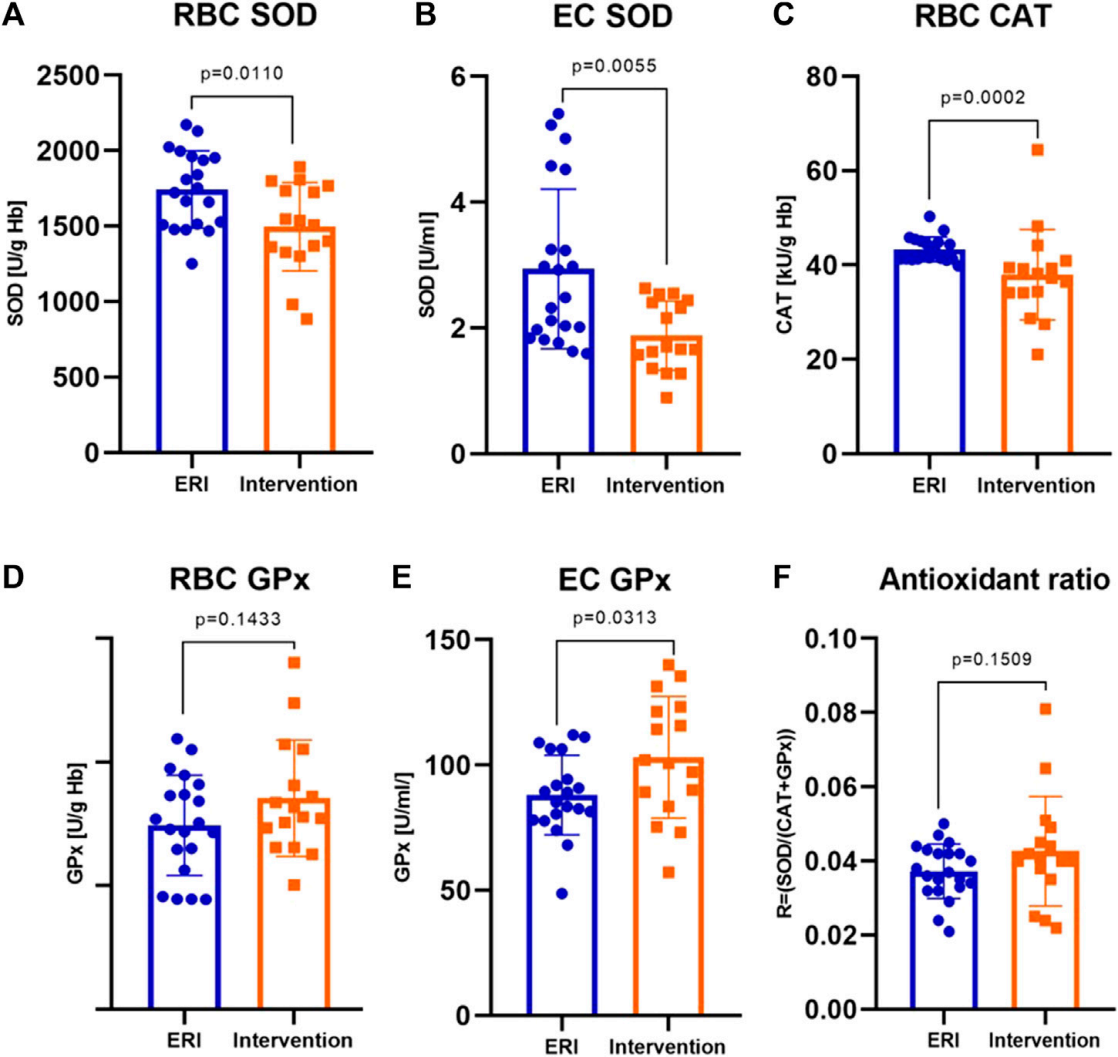
Erythrocyte and extracellular antioxidants activity in ERI and intervention group. The following enzymes showed differences in activities: erythrocyte superoxide dismutase, RBC SOD, *p* = 0.0110 **(A)**, extracellular superoxide dismutase, EC SOD, *p* = 0.0055 **(B)**, erythrocyte catalase, RBC CAT, *p* = 0.0002 **(C)** and extracellular glutathione peroxidase, EC GPx, *p* = 0.0313 **(E)**. The changes in erythrocyte glutathione peroxidase activity, RBC GPx **(D)** and antioxidant ratio **(F)** were not statistically significant (*p* = 0.1433 and *p* = 0.1509, respectively). Data were presented as mean ± SEM (at 95% CI) except for CAT activity, where data was presented as median with interquartile range (depending on the data distribution) (ERI group, n = 20, Intervention group, n = 16, statistical significance, *p* < 0.05).

**FIGURE 2:**
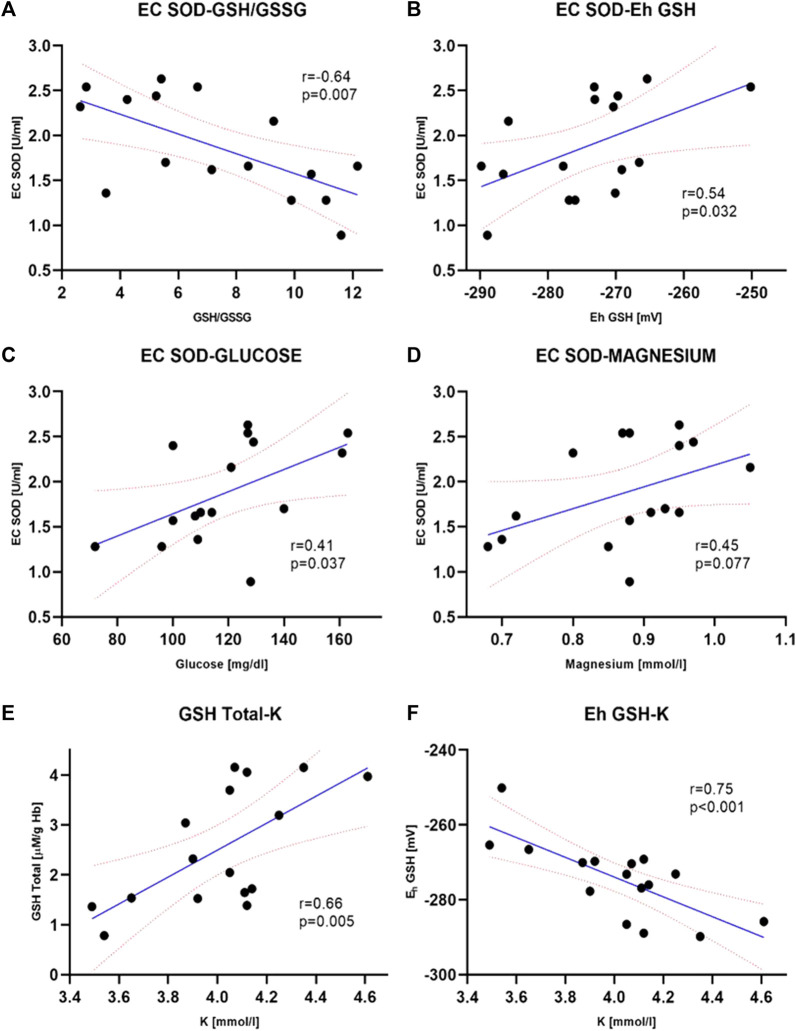
Selected clinically significant correlations between extracellular superoxide dismutase, EC SOD activity and biochemical parameters–GSH/GSSG, *p* = 0.007 **(A)**, GSH, *p* = 0.032 **(B)**, plasma glucose concentration, *p* = 0.037 **(C)**. The correlation between EC SOD and plasma magnesium concentration **(D)** was not statistically significant (*p* = 0.077) however, there is a noticeable tendency to increase enzyme activity with increasing magnesium concentration. Correlations between total GSH concentration (GSH + GSSG) **(E)** and E_h_ redox potential **(F)** in erythrocytes and potassium level in plasma are also shown. Both were statistically significant (*p* = 0.005 and *p* < 0.001, respectively) and are clinically important. The values of correlation coefficients and *p*-values are also given on the graphs (ERI group, n = 20, Intervention group, n = 16, statistical significance, *p* < 0.05).

Main antioxidant enzyme activity in patients with VA was significantly altered compared to the ERI group. Erythrocyte SOD activity was significantly lower by about 15% (1,496.3 ± 73.0 vs. 1722.7 ± 57.9 U/g Hb, Figure 1A). A similar but more evident change was present in plasma samples, where the decrease in activity of EC SOD was about 36% (1.68 ± 0.95 vs 2.40 ± 1.33 U/mL, Figure 1B). This indicates a reduced dismutation ability of the superoxide anion radical (O_2_^−^) to H_2_O_2_. With an increase in EC SOD activity, a moderate decrease in GSH/GSSG ratio and an increase in Eh GSH (more positive) was observed (Figures 2A,B). It is associated with an increase in the intensity of oxidative stress. An inverse relationship was observed for glucose and magnesium concentration (*p* = 0.037 and *p* = 0.077, respectively) (Figures 2C,D). CRP concentration seems to have a moderate effect on SOD activity in erythrocytes. There is a tendency to increase CRP with a simultaneous decrease in SOD activity (border of statistical significance, *p* = 0.089), which may point to the influence of inflammation on the redox status (Figure 5).

CAT activity in patients with arrhythmia was significantly lower by about 11% compared to patients from the ERI group (37.7 ± 6.00 vs. 42.5 ± 3.80 kU/g Hb, Figure 1C). The above results (SOD and CAT) indicate an accumulation of H_2_O_2_ due to decreased CAT activity, which may result from inhibition by O_2_^−^.

Erythrocyte and EC GPx activity was higher in the intervention group, but only differences in EC GPx were statistically significant. The mean enzyme activity in erythrocytes was about 13% higher (170.8 ± 11.8 vs. 148.9 ± 9.1 U/g Hb, Figure 1D), and the average values in plasma were by about 16% higher in the intervention group (103.1 ± 6.1 vs. 86.7 ± 3.5 U/g Hb, Figure 1E) than in control group. This change may be due to a decrease in SOD and CAT activity and the accumulation of H_2_O_2_.

### 3.2 Antioxidant de Haan ratio

We examined the de Haan ratio (Figure 1F) to check whether the antioxidant defense is sufficient. The increased ratio indicates the higher activity of SOD in relation to CAT and GPx, which may suggest a higher rate of H_2_O_2_ synthesis or accumulation in relation to its decomposition by GPx and is more appropriate in understanding the redox status and free radicals impact on redox balance than the absolute activities of enzymes themselves. The ratio was calculated using the following equation: R = SOD/CAT + GPx. An imbalance in relation of SOD to CAT and GPx results in the accumulation of H_2_O_2_, which may participate in the Fenton reaction, forming hydroxyl radicals. This ratio was insignificantly higher in the intervention than in the control group (mean 0.043 ± 0.01 vs. 0.037 ± 0.01).

### 3.3 Analysis of concentration of total glutathione, oxidized glutathione and redox potential

**FIGURE 3:**
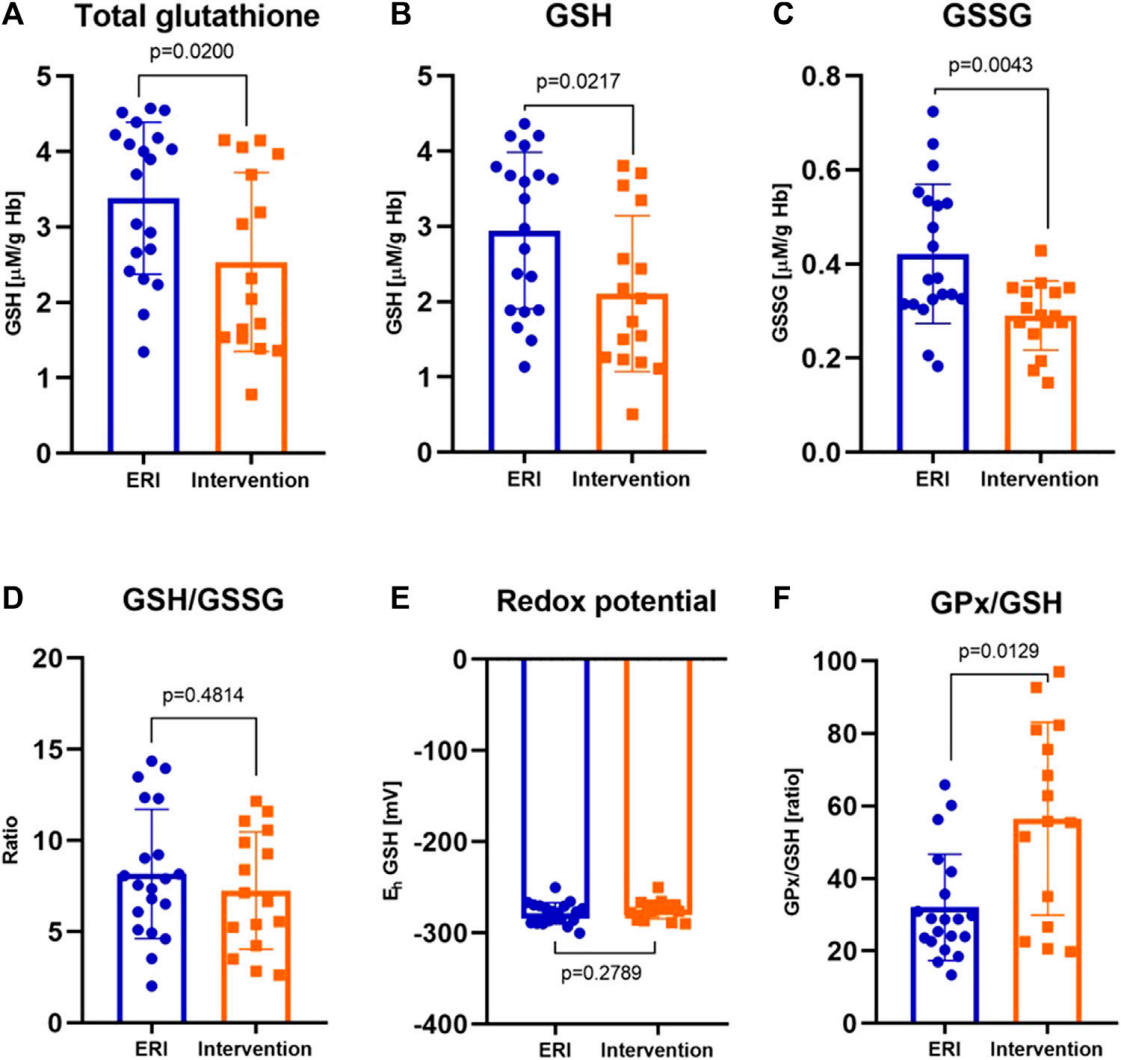
The concentration of total glutathione in erythrocytes **(A)**, reduced glutathione, GSH **(B)**, oxidized glutathione, GSSG **(C)** were significantly lower in patients after ICD/CRT-D intervention (*p* = 0.0200, *p* = 0.0217 and *p* = 0.0043, respectively) Reduced to oxidized glutathione ratio, GSH/GSSG **(D)** and the redox potential, E_h_ **(E)** were similar in both groups (*p* = 0.4814 for GSH/GSSG and *p* = 0.2789 for redox potential). The glutathione peroxidase to reduced glutathione ratio, GPx/GSH **(F)** was definitely greater after the intervention (*p* = 0.0129). Data were presented as median with interquartile range except for GSH and E_h_, where mean ± SEM values (at 95% CI) are shown (depending on the data distribution) (ERI group, n = 20, Intervention group, n = 16, statistical significance, *p* < 0.05).

In all patients from the study group, a decrease in the concentration of all forms of glutathione was observed compared to the control ERI group (Figure 3). Total GSH concentration was lower by about 27% (2.54 ± 0.30 vs. 3.46 ± 0.22 μM/g Hb, Figure 3A), GSH by about 28% (2.11 ± 0.26 vs. 2.95 ± 0.24 μM/g Hb, Figure 3B), and GSSG by about 15% with border significant (0.35 ± 0.29 vs. 0.41 ± 0.37 μM/g Hb, Figure 3C). The GSH/GSSG ratio, which represents a dynamic balance between oxidants and antioxidants, was the same in both groups indicating no glutathione redox imbalance. However, it seems that in patients after ICD/CRT-D intervention, this ratio may be slightly shifted in favor of GSSG (median 7.26 ± 0.81 in the study group vs. 8.24 ± 0.83 in the control group, Figure 3D). A small insignificant difference in the redox potential for glutathione was observed. In the intervention group, it became more positive, i.e., more oxidizing. The average difference was 4.2 mV. There is an apparent relationship between the concentration of total glutathione and the plasma concentration of potassium and E_h_ (Figure 2E). The increase in total and reduced glutathione is accompanied by the accumulation of potassium and, at the same time, a decrease in E_h_ potential (more negative, i.e., more reducing, Figure 2F).

### 3.4 GPx and glutathione

GPx is an important modulator of the balance between GSH and GSSG. We observed a significant increase in GPx to GSH ratio in patients with ICD intervention (median 55.77 ± 54.39 vs. 28.9 ± 20.26 in the control group, Figure 3F). GPx recycling is dependent on GSH availability. We suggest that the decrease in the GSH concentration was probably due to the high GPx activity (and accumulation of H_2_O_2_) since it represents its coenzyme and high rate of GSH oxidation.

### 3.5 Reactive oxygen and nitrogen species

**FIGURE 4:**
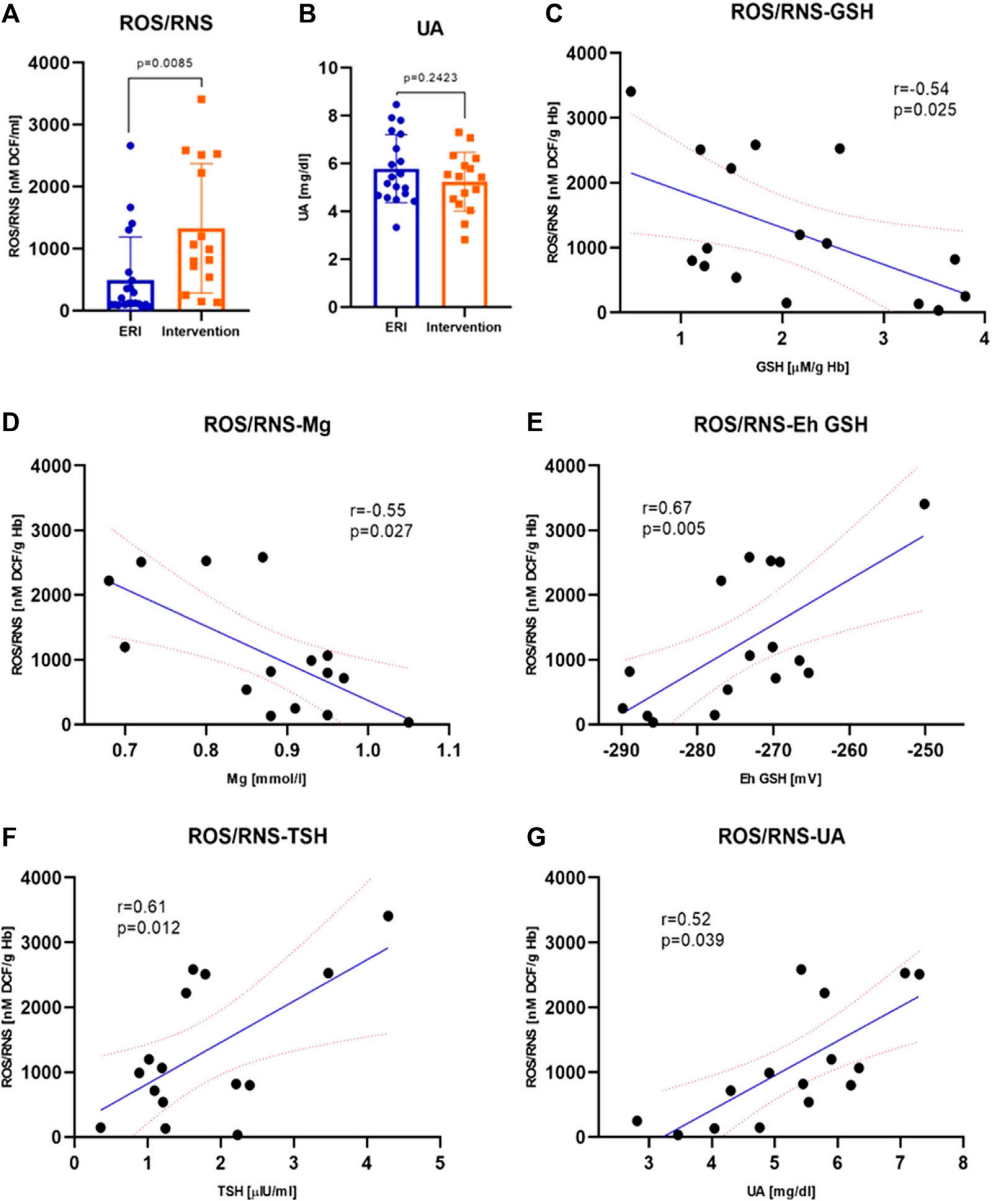
Plasma ROS/RNS concentration was higher after intervention (*p* = 0.0085); data were presented as median with interquartile range **(A)**. Plasma uric acid concentration was similar in both groups (*p* = 0.2423); data were presented as mean ± SEM at 95% CI **(B)** Correlations between ROS/RNS plasma concentration and selected biochemical parameters–GSH **(C)**, Mg **(D)**, E_h_ GSH **(E)**, TSH **(F)** and UA **(G)** were statistically significant (*p* = 0.025, *p* = 0.027, *p* = 0.005, *p* = 0.012 and *p* = 0.039, respectively). The values ​​of correlation coefficients and *p*-values ​​are given also on the graphs (ERI group, n = 20, Intervention group, n = 16, statistical significance, *p* < 0.05).

The concentration of ROS/RNS was significantly higher in the intervention group. The median of the total concentration of H_2_O_2_, NO, ROO^•^, and ONOO^−^ is 987.2 ± 1973.2 in the study vs. 245.9 ± 856.3 nM DCF/mL in the control group, Figure 4A. It is a difference of about 300%. This indicates increased ROS/RNS synthesis or accumulation in the perishock period. The most significant and interesting relationships were obtained for ROS/RNS, EC SOD, potassium, magnesium, UA and thyroid stimulating hormone (TSH) concentration. ROS/RNS concentration was negatively, moderately correlated with the concentration of GSH and magnesium (Figures 4C,D). The plasma concentration of ROS/RNS moderately increased with the redox E_h_ potential, concentration of TSH and UA (Figures 4E–G).

### 3.6 Uric acid concentration in plasma

There is evidence that UA can act as an antioxidant (mainly in the plasma) or prooxidant (mainly in the cell). We showed no significant differences in UA concentration between the study and control group, but UA in the control group showed a slightly lower concentration which may indicate a lower antioxidant capacity of plasma (Figure 4B).

### 3.7 The diagnostic value of examined redox status parameters

**FIGURE 5:**
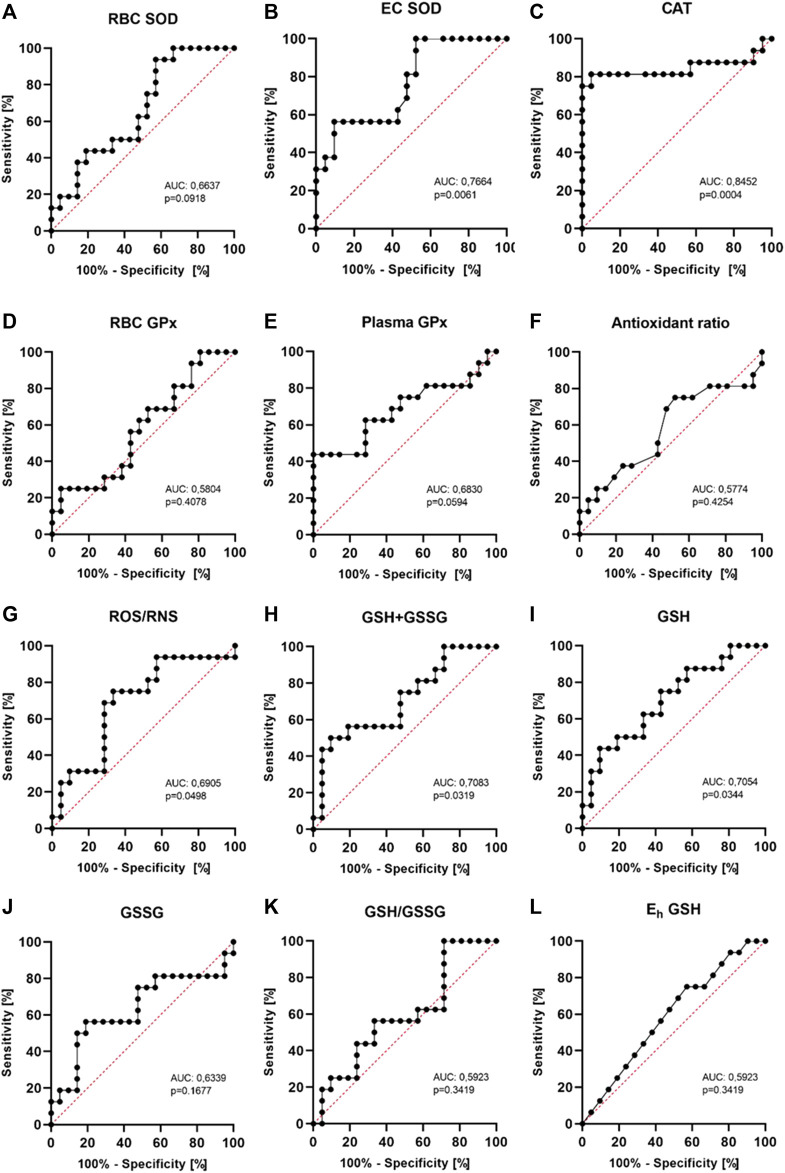
Receiver operating characteristics (ROC curves) for the analyzed markers. The area under the curve (AUC) and *p* values are shown in the individual graphics. The most diagnostically useful indicator of redox imbalance may be CAT activity with sensitivity of 81.3% and specificity of 95.2% (AUC 0.8452, 95% CI 0,6844−1,000, *p* = 0.0004) with a cut-off value of 41 kU/g Hb.

In the current study on a small group of patients, it seems that the most diagnostically useful indicator of redox imbalance in patients after ICD intervention may be CAT activity with sensitivity of 81.3% and specificity of 95.2% (AUC 0.8452, 95% CI 0,6844–1,000, *p* = 0.0004) with a cut-off value of 41 kU/g Hb (Figure 5). ROS/RNS has less sensitivity of 75.0% and specificity of 66,7% with a cut-off value of 511 nM (AUC 0.6905, 95% CI 0,5142–0,8668, *p* = 0.0498). Other parameters seem less useful. Especially RBC SOD, RBC GPx, antioxidant ratio, GSH concentration, GSH/GSSG ratio and Eh GSH have low diagnostic value. EC SOD and plasma GPx require further research. CAT and ROS/RNS can help in monitoring the metabolic response of patients.

### 3.8 Other important correlations

**FIGURE 6:**
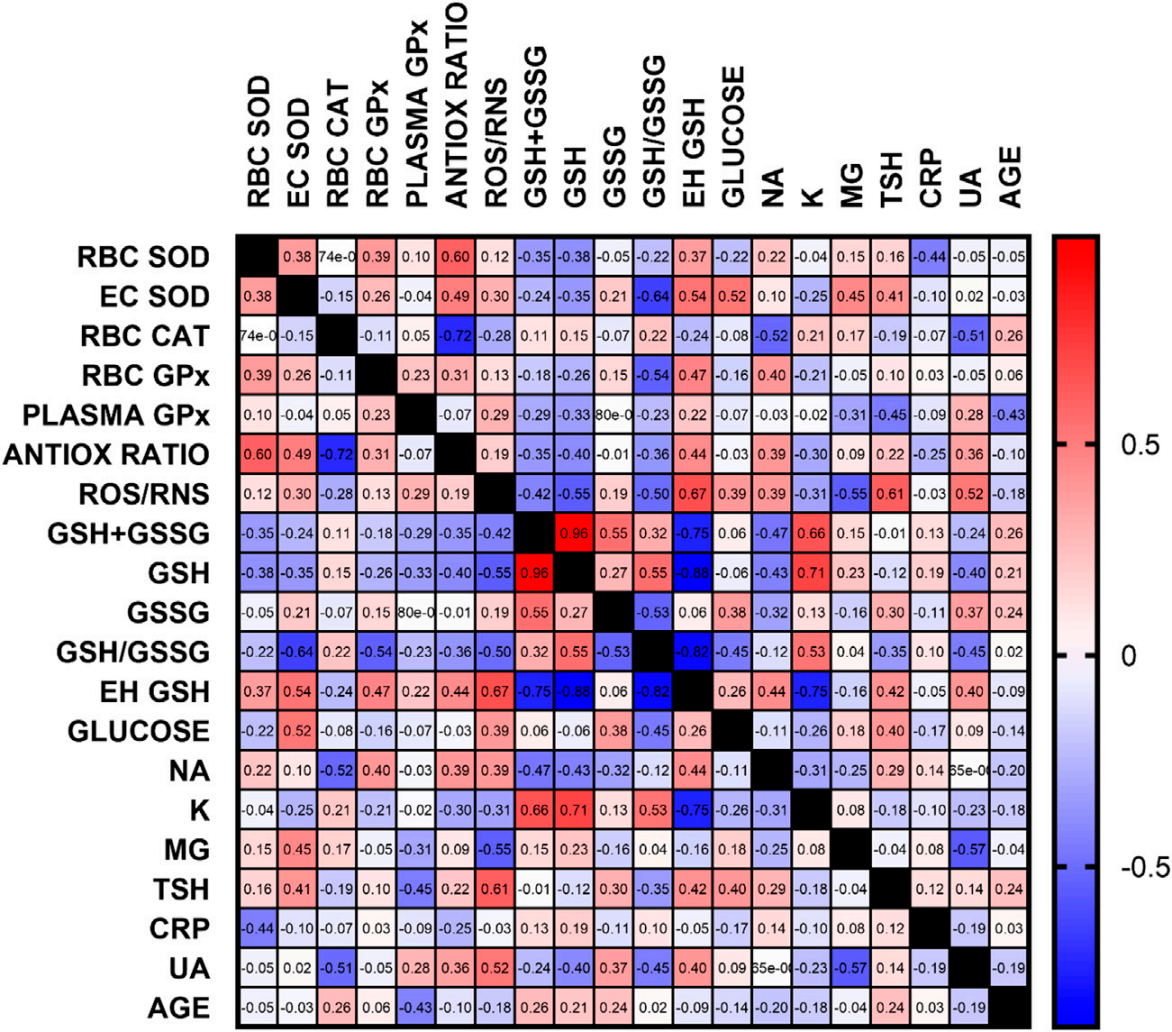
Correlation matrix of all oxidative stress and additional, routinely determined, biochemical parameters. Data are presented as correlation coefficients, regardless of the *p*-value. The most important correlations were found for antioxidant parameters, glutathione, ROS/RNS, ion concentration, TSH and UA (RBC–erythrocytes, GSH–reduced glutathione, GSSG–oxidized glutathione, E_h_–redox potential, Na–sodium, K–potassium, Mg–magnesium, TSH–thyroid stimulating hormone, CRP–C reactive protein, UA–uric acid)

Apart from the previously discussed relationships, the negative correlation between the concentration of uric acid and the activity of catalase, reduced to oxidized glutathione ratio and magnesium concentration seems important. This may indicate the need for close monitoring of UA concentration in patients with ventricular arrhythmias. All correlations are presented in Figure 6, and all statistics of biochemical parameters are presented in Table 3. Despite the small study group, the demonstrated relationships are significant from a clinical point of view, especially regarding ion balance, TSH concentration and the presence of inflammation.

**TABLE 3:** Statistics of all biochemical parameters.

		Mean	SEM	Min	Max	Median	Q1	Q3	*p*-value
**Erythrocyte SOD [U/g Hb]**	ERI	1722.7^a^	57.9	1,251.2	2172.1	1721.1	1,510.2	1952.9	**0.0110** ^**a**^
	INT	1,496.3^a^	73.0	884.9	1892.8	1,522.5	1,343.7	1750.2	
**Plasma SOD [U/mL]**	ERI	2.82	0.27	1.59	5.41	2.40^b^	1.91	3.24	**0.0089** ^**b**^
	INT	1.88	0.14	0.89	2.63	1.68^b^	1.47	2.42	
**Erythrocyte CAT [kU/g Hb]**	ERI	45.2	2.0	39.8	81.7	42.5^c^	41.3	45.1	**0.0002** ^**c**^
	INT	37.9	2.4	21.1	64.5	37.7^c^	34.1	40.1	
**Erythrocyte GPx [U/g Hb]**	ERI	148.9^d^	9.1	88.9	218.8	148.2	121.0	177.8	0.1433^d^
	INT	170.8^d^	11.8	100.7	280.3	159.4	138.8	195.8	
**Plasma GPx [U/mL]**	ERI	86.7^e^	3.5	48.7	111.2	85.2	78.1	94.3	**0.0209** ^e^
	INT	103.1^e^	6.1	57.2	139.9	101.3	86.3	122.2	
**Antioxidant ratio**	ERI	0.037^f^	<0.01	0.021	0.050	0.037	0.033	0.042	0.1509^f^
	INT	0.043^f^	<0.01	0.022	0.081	0.041	0.037	0.047	
**Erythrocyte GSH + GSSG [µM/g Hb]**	ERI	3.46	0.22	1.34	4.57	3.89^g^	2.66	4.22	**0.0200** ^**g**^
	INT	2.54	0.30	0.78	4.15	2.18^g^	1.53	3.83	
**Erythrocyte GSH [µM/g Hb]**	ERI	2.95^h^	0.24	1.14	4.37	3.17	1.88	3.74	**0.0217** ^h^
	INT	2.11^h^	0.26	0.51	3.81	1.89	1.25	2.96	
**Erythrocyte GSSG [µM/g Hb]**	ERI	0.41	0.03	0.18	0.72	0.37^i^	0.31	0.53	**0.0043** ^**i**^
	INT	0.35	0.05	0.15	0.87	0.29^i^	0.25	0.35	
**GSH/GSSG ratio**	ERI	8.24	0.83	2.03	14.36	7.90^j^	5.11	12.30	0.4814^j^
	INT	7.26	0.81	2.62	12.16	6.90^j^	4.74	10.23	
**E**_**h**_ **potential [mV]**	ERI	−274.3^k^	2.5	−289.8	−250.1	−273.1	−281.8	−269.4	0.2789^k^
	INT	−278.5^k^	2.6	−300.1	−250.1	−277.3	−287.8	−270.2	
**Plasma ROS/RNS**	ERI	584.9	161.4	57.8	2663.0	245.9^L^	103.5	959.8	**0.0085** ^**L**^
	INT	1,326.6	269.9	130.3	3409.3	987.2^L^	538.0	2511.2	
**Uric acid**	ERI	5.78^m^	0.33	3.33	8.46	5.44	4.66	7.23	0.2423
	INT	5.24^m^	0.31	2.81	7.30	5.44	4.41	6.06	

## 4 Discussion

Many studies in cellular and animal models have shown an association between redox imbalance and the occurrence of cardiac arrhythmias. The influence of oxidative stress on arrhythmias in patients with HF may be significant since it plays a critical role in the pathogenesis of HF (Tsutsui et al., 2011; van der Pol et al., 2019). So far, most researches has focused on supraventricular arrhythmias, such as AF (Lin et al., 2010; Korantzopoulos et al., 2018; Hsu et al., 2023). There is a lack of data on ventricular arrhythmias, particularly in patients with ICD/CRT-D. In these patients, the electrical impulses may lead to cardiac damage (e.g., by cytolysis or electroporation), to generation of ROS/RNS and increase the risk of arrhythmias in the perishock period (Trouton et al., 1989; Clementy et al., 2021). It has been shown that statins affecting oxidative stress and reducing oxidation may reduce the frequency of ICD therapy (Bloom et al., 2010). Unfortunately, it is unclear whether an increase in oxidative stress is an arrhythmogenic factor leading to ICD/CRT-D interventions or is mainly its effect. Our study showed a significant systemic redox imbalance up to 6 h after electrical shock and its relation with ionic, TSH and UA homeostasis.

Antioxidant enzymes are proteins involved in ROS catalytic transformation and neutralization into stable, nontoxic molecules. We showed, that after ICD/CRT-D shock, there was an evident decrease in SOD and CAT activity, an increase in erythrocyte and EC GPx activity, and ROS/RNS concentrations. It indicates intensive oxidative stress in the perishock period in patients with life-threatening arrhythmias. This is in line with new proteomic studies in a sheep model that showed lower expression of proteins involved in ROS metabolism, oxygen transport, or respiratory chain proteins in the heart tissues collected from the ventricles (Bodin et al., 2020). It was also shown that an increase in proteins involved in the detoxication of ROS/RNS may arise from an adaptive response to oxidative stress (Bodin et al., 2020). Our observations indicate not only local myocardial tissue disturbances but also systemic ones, visible in changes in extracellular enzyme activity. They can have a long-term effect and can be determined using non-invasive methods.

SOD is a major cellular and extracellular antioxidant metalloenzyme that catalyzes the dismutation of superoxide radical (O_2_^•-^) to hydrogen peroxide (H_2_O_2_) and oxygen. Its decrease leads to a reduced dismutation ability and accumulation of the O_2_^•-^ which may also be due to inhibition by high concentration of H_2_O_2_ (Kono and Fridovich, 1982) caused by the revealed 11% decrease in CAT activity. The greatest decrease of EC SOD observed in plasma may facilitate its practical use in routine diagnostics and indicates both intracellular and extracellular changes. CAT breaks down H_2_O_2_ into oxygen and water. A decrease in CAT activity, arised from inhibition by O_2_^•-^(which is consistent with a 36% decrease in EC and 15% RBC SOD), affects accumulation of H_2_O_2_ (Kono and Fridovich, 1982). GPx reduces H_2_O_2_ to water while oxidizing GSH. Its increase may be an effect of decrease in SOD and CAT activity and accumulation of H_2_O_2_. An imbalance in relation of SOD to CAT and GPx results in the accumulation of H_2_O_2_, which participate in the Fenton reaction, resulting in the formation of hydroxyl radicals (OH^•^) (H_2_O_2_ and OH^•^ are observed in our results as an increase in ROS/RNS concentration). Up to 50% SOD deficiency in patients with supraventricular and ventricular arrhythmias demonstrates the involvement of oxidative stress in cardiac arrhythmic pathogenesis (Eznă et al., 2017). A decrease in SOD activity can lead to O_2_^•-^ accumulation, affect H_2_O_2_ concentration, misregulate many redox-sensitive pathways and may cause myocardial remodeling and failure (Siwik et al., 1999; Yin. et al., 2018). Studies on animals have shown a reduction in reperfusion arrhythmias after SOD use and has indicated that arrhythmias may be due to O_2_^•-^ (Nejima et al., 1989; Ohoi and Takeo, 1996). The EC SOD isoform is also crucial because it modulates O_2_^•-^ levels in the vasculature (Yin. et al., 2018), which plays a critical role in the heart for normal cardiac function protecting the myocardium from oxidant-induced fibrosis (Kliment et al., 2009). Bezna et al. indicated that GPx, which catalyzes the reduction of a wide range of hydroperoxides from nonorganic H_2_O_2_ to complex organic hydroperoxides at the expense of GSH, may also be a specific biomarker of oxidative stress in young people with cardiac arrhythmias and may be involved in arrhythmogenic electrochemical processes (Bezna et al., 2022). It was noted that statins have a significant antioxidant effect through the upregulation of SOD and GPx and may have a protective antioxidant effect (Zinellu and Mangoni, 2021). An increase in SOD, CAT and GPx activities by the use of quercetine can exert beneficial effects on arrhythmias by affecting ion channels, Ca^2+^ homeostasis, gap junction, suppressing cardiac fibrosis or inflammation and by inhibition or regulation of critical signaling pathways involved in arrhythmias, such as TGF-β/Smad, NF-κB, and PI3K/AKT (Zhou et al., 2022). A noticeable but statistically insignificant (*p* = 0.1509) increase in antioxidant ratio in our study may suggest a higher rate of H_2_O_2_ synthesis or accumulation in relation to its decomposition by GPx.

ROS/RNS play a fundamental function in cell homeostasis in the heart, but its excess can affect the late sodium current and cause an arrhythmia, such as VF (Qu et al., 2011). They can also affect the function of cardiac Ca^2+^ channels, cause impaired ventricular contractility and after-depolarization-mediated triggering cardiac arrhythmias (Choudhary and Dudley, 2002). The observed in our study increase in concentration of ROS/RNS in the perishock period indicates increased oxidative stress, probably due to H_2_O_2_. The electrical shock of an ICD/CRT-D can cause an increase in ROS/RNS concentration, but it can also be its result. Transthoracic electrical shock can generate free radicals, depress Ca^2+^-pumping function and cause myocardial dysfunction (Jones and Narayanan, 1998; Oltman et al., 2003; Clementy et al., 2021). A study in dogs has shown that direct current (DC) during defibrillation shocks can generate ascorbate free radicals (ASC) (Caterine et al., 1996). A peak of ASC increase of 14% was seen 5–6 min after 100 J epicardial shocks and 7% after 200 J transthoracic shock, wherein multiple shocks did not result in a greater ASC production, and the administration of SOD and CAT before shock resulted in a reduction of ASC concentration (Caterine et al., 1996). Trouton et al. suggested that DC shocks lead to mitochondrial dysfunction, which generates, e.g., superoxide production (Trouton et al., 1992). In our study, samples were not collected immediately after the shock. Despite this, we observe significant redox-balance changes. The free radical concentration peak occurs a few minutes after discharge (Caterine et al., 1996; Zhang et al., 2003). Therefore, we speculate that redox imbalance may have been present even before the intervention of the ICD/CRT-D.

Magnesium administration before defibrillation might be cardioprotective and reduce the oxygen free radicals generated by DC shocks. Total radical flux can be reduced by about 72% (Zhang et al., 2003). Matkovics et al. reported that magnesium treatment resulted in up to 10%–50% increase in the activation of SOD and CAT as well as in the concentration of GSH (Matkovics et al., 1997). In our study, the concentration of ROS/RNS was lower, and the activity of EC SOD was the higher the higher the magnesium concentration was observed. This confirms previous observations and the validity of magnesium control in patients with ventricular arrhythmias. ROS level is also related to TSH, whose increase and hypothyroidism is associated with increased oxidative stress response (Chakrabarti et al., 2016). Thyroid cells release enzymes that catalyse ROS generation, therefore the increase in TSH level may be a factor associated with increased oxidative stress and risk of arrhythmias. Previously thought that hyperthyroidism directly affects the cardiovascular system (Ahmad et al., 2022). An increase of TSH may affects the intensity of redox disturbances, visible as a significant increase in ROS/RNS concentration and a slight increase in EC SOD activity, GSSG concentration, E_h_ potential, and a decrease in plasma GPx activity.

We also observed significant changes in glutathione concentration. GSH represents the largest capacity thiol buffer in mammalian cells and plays a critical role in the detoxification of H_2_O_2_ via GPx. Also, mitochondrial GSH plays a critical role in defense against the ROS and is involved in the development of arrhythmias. Severe oxidative and nitrosative stress leads to lower GSH levels in cells and an increased turnover of the GSH/GSSG cycle (Jones, 2002). We showed that total glutathione, GSH and GSSG were lower in the intervention compared to the control group. GSH oxidation leads to an accumulation of ROS in the mitochondria of cardiomyocytes and causes electromechanical dysfunction, which depends on the mitochondrial energy state (Brown et al., 2010). The imbalance of GSH/GSSG can result in a collapse of mitochondrial inner membrane potential (ΔΨm) and cardiac arrhythmias (Brown et al., 2010) but may also reflect changes in redox signaling and control (Jones, 2002). Simultaneous decrease of both forms - GSH and GSSG did not cause GSH/GSSG imbalance (observed in our study) but decreased the antioxidant capacity of the GSH system. There is a very small tendency to reduce the GSH E_h_ potential (*p* = 0.2789), which becomes more positive, i.e., more oxidizing. Maintaining the balance of GSH and GSSG levels protects the heart from arrhythmias (Frasier et al., 2011). It is clinically relevant to maintain normal potassium concentration, which significantly positively correlates with GSH, and its higher level leads to a decrease in E_h_ GSH.

Uric acid levels in HF patients closely correlate with disease severity and are an important prognostic factor (Ng et al., 2023). UA is a product of xanthine oxidoreductase and has both antioxidant and pro-oxidant properties. UA metabolism runs with the accompanying production of ROS and might contribute to increased oxidative stress. UA may inhibit CAT activity and promote prooxidative processes. In our study, examined patients did not differ in the concentration of UA. However, the increase in UA concentration correlated with an increase in ROS/RNS, GSSG and E_h_ GSH, while GSH, GSH/GSSG and magnesium concentration were lower. Therefore, UA may be associated with a higher risk of arrhythmias in patients with HF. Magnesium (especially MgSO_4_) has been used to treat different types of arrhythmias for several decades and ensuring an adequate magnesium concentration can positively affect lowering UA and ROS/RNS concentration. Hypomagnesemia is commonly observed in HF and is associated with oxidative stress and suppressing the antioxidant defense system (e.g., SOD, CAT, GSH) (Liu and Dudley, 2020). Magnesium reduces ROS generated by DC shocks and may be cardioprotective during defibrillation/cardioversion (Zhang et al., 2003). Maintaining higher concentrations of magnesium may promote the excretion of UA.

Figure 6 clearly indicates the existence of relationships not only between oxidative stress parameters, but also between the concentrations of basic ions, thyroid stimulating hormone and uric acid concentration. This is very important information for clinicians who should simultaneously treat comorbidities in a patient with arrhythmia or correct biochemical parameters that may be related to the severity of oxidative stress. These may include electrolyte disorders, diabetes, thyroid disease and inflammation.

For the first time we showed that oxidative stress may be significant in VAs and ICD/CRT-D interventions. A broad, innovative view of oxidative stress and its role in cardiovascular diseases may allow for better treatment of arrhythmias and reduce ICD interventions.

## 5 Conclusion

Oxidative stress has been linked to the development of cardiac ventricular arrhythmias. Pathophysiological processes may be related to an ion channels function, activity of metalloproteinases, cardiac fibrosis and myocardial remodeling - a major pathological mechanism in HF which various ROS-related pathways can regulate. Monitoring the severity of redox imbalance and the response of antioxidant systems may be important in patients with HF and ICD/CRT-D. It is an entirely new look at the possibility and chances of preventing cardiac ventricular arrhythmias. Better monitoring of basic parameters, e.g., ion concentration, TSH and UA, and the use of antioxidants available in the diet may prove particularly useful. However, this requires further multicenter studies on a larger group of patients.

## Data Availability

The raw data supporting the conclusion of this article will be made available by the authors, without undue reservation.
